# Barriers in Access to Healthcare Services in Greece Post-COVID-19: Persisting Challenges for Health Policy

**DOI:** 10.3390/healthcare13151867

**Published:** 2025-07-30

**Authors:** Kyriakos Souliotis, Christina Golna, Agni Baka, Aikaterini Ntokou, Dimitris Zavras

**Affiliations:** 1 School of Social and Political Sciences, University of Peloponnese, 20100 Corinth, Greece; 2 Health Policy Institute, 15123 Marousi, Greece; pm@healthpolicyintitute.eu (C.G.); ab@healthpolicyinstitute.eu (A.B.); kn@healthpolicyinstitute.eu (A.N.); 3 Department of Public Health Policy, School of Public Health, University of West Attica, 11521 Athens, Greece; dzavras@uniwa.gr

**Keywords:** healthcare access, health policy, barriers, health services accessibility, socioeconomic factors, Greece

## Abstract

**Background/Objectives**: Access to health services is often limited due to socio-economic and organizational determinants of health systems, which lead to increased unmet healthcare needs. This study aimed to identify access barriers for the general population in Greece, including those that may have emerged following the COVID-19 pandemic. **Methods**: This was a cross-sectional survey of 1002 Greek citizens. A questionnaire regarding socio-demographics, healthcare utilization, and access to health services was used. Interviews took place between October and November 2022. **Results**: Of 837 participants who used health services in 2022, 82.6% had a medical consultation, 80.6% took diagnostic tests, and 63.6% visited a pharmacy for pharmaceuticals. Of those having a medical consultation, 33.1% did so at an NHS health unit, while 75% of the participants taking diagnostic tests visited a contracted private laboratory. Out of the 135 participants requiring hospitalization, 62% were hospitalized in a public hospital, while 85% of the participants requiring pharmaceuticals visited a private pharmacy. Access barriers in the past year were reported by 48% of the participants requiring a medical consultation, 34% of the participants requiring diagnostic tests, and 40% of the participants requiring hospitalization. The most common barriers were long waiting times and financial constraints. The main barrier to accessing pharmaceuticals was the availability and administration of the product. **Conclusions**: The identified healthcare access barriers highlight the vulnerabilities of the current health system in Greece, which were further exposed during the COVID-19 pandemic crisis. Addressing socioeconomic factors that are considered key access indicators should be the focus of future health policy initiatives.

## 1. Introduction

The concept of quality in healthcare is complex and multidimensional, and has been defined in various ways [1]. Following a disaggregated approach, quality of health services should be evaluated according to individual components of the health system structure, processes, and outcomes [1,2,3]. Access to health services, relevance to the needs of individuals and the community, fairness, effectiveness for individual patients, and social acceptability constitute examples of these components [4,5].

Accessibility in healthcare consists of different dimensions, including physical access in terms of geographical distance and availability of healthcare facilities, organizational access in terms of availability of appointments and health professionals, and financial access in terms of affordable health services [2,6]. Equity considerations in access, defined according to whether all individuals in a population have access to the healthcare they need, should also be taken into account [1]. In every case, accessibility in healthcare is considered internationally a significant quality indicator in health services evaluation. It should be noted, however, that self-reported “need for health care” typically concerns the individuals’ own perception according to their symptoms or health concerns, and the perceived unmet need indicates situations where individuals did not receive the medical care they felt they needed [7,8]. In that sense, the self-reported unmet healthcare needs reflect barriers in receiving the necessary healthcare services.

Access to health services is often characterized by considerable barriers, such as high costs, shortage or lack of healthcare facilities and staff, geographical disparities, and lack of social support. The outbreak of two global crises, the 2008 economic crisis and the 2020 COVID-19 pandemic crisis, has further highlighted these vulnerabilities in healthcare systems worldwide. Both crises had stimulated changes in healthcare resource management and the implementation of policies that shifted the financial burden of healthcare to households. These changes adversely affected access to health services and led to an increase in unmet healthcare needs [9,10,11]. In fact, between 2021 and 2022, about half of the countries in the European Union (EU) showed increasing rates of self-reported unmet healthcare needs [12].

Healthcare in Greece consists of a mixed public–private healthcare system, where universal coverage is provided by the National Health System (NHS) to all citizens via social insurance (fund) contributions and taxation [13,14]. Eligibility for free public healthcare generally requires an active insurance status, although reforms following the 2008 economic crisis and the COVID-19 pandemic have expanded access to essential care for vulnerable populations, including uninsured individuals [15,16]. Since 2011, the public health system covers primary and secondary care, pharmaceuticals, and preventive services via a single entity named the National Organization for Health Care Services (EOPYY), which also enters into contracts with private healthcare facilities [17]. However, significant co-payments are often required for pharmaceuticals and medical consultations to specialists, while out-of-pocket expenses remain high, partly due to inefficiencies in the system and informal payments [14,18,19]. For instance, private health services are increasingly used because of long waiting times and differences in perceived quality of care, and are generally not reimbursed by the public sector.

In Greece, reduced healthcare resources during the economic crisis have posed barriers to healthcare access, mainly due to increased demand, waiting times, and co-payments, and a decline in the ability to cover informal payments [20,21]. Indeed, following the economic recession, unmet healthcare needs have increased in Greece, and this trend is still present [22,23]. Additionally, the COVID-19 pandemic has led to increased access barriers and supply restrictions by the Greek healthcare system, which resulted in a significant decrease in health services utilization [24]. Specifically, healthcare access disruption during the COVID-19 pandemic, on the one hand, was linked to demand side factors, such as patients’ inability to access healthcare services due to transport restrictions during the lockdowns, reduced healthcare-seeking behavior due to fear or to preserve health system capacity, and deepening poverty that may limit the ability of households to cover out-of-pocket costs. On the other hand, it was linked with supply-side factors such as supply chain interruptions of essential medicines and other health products, diversion of health resources from other services to the COVID-19 response, and paused provision of certain services to curb the spread of the virus [25].

Self-reported unmet healthcare needs due to costs, geographical distance, or long waiting lists in Greece have remained consistently above the EU average in the past years. In fact, Greece experienced the highest increase in the rate of unmet needs for healthcare between 2021 and 2022 (by 2.6%) compared to an average EU increase of 0.2% [12]. Low-income households reported three times higher rates of unmet healthcare needs compared to high-income groups, indicating costs as a main driver of unmet needs. Additionally, according to a 2022 Eurostat report regarding unmet healthcare needs, 16.7% of Greek citizens aged 16 years or above reported unmet needs for medical examination due to financial constraints, long waiting times, or geographical distance, while 28% of those reported being at risk of poverty [26].

Several previous studies have investigated barriers to accessing health services in Greece [20,27,28,29,30,31]. However, these studies focused on specific patient populations, as for instance patients with multiple sclerosis or cancer, and specific time periods, such as the era following the economic crisis in Greece. The identified healthcare access barriers were similar between studies, and highlighted the relevance of obstacles such as high costs, long waiting lists, and distance in meeting healthcare needs for these patient groups. Our study aims to confirm these findings in relation to the general population in Greece and evaluate the presence of additional determinants affecting the level of unmet healthcare needs following the outbreak of the COVID-19 pandemic.

## 2. Materials and Methods

A cross-sectional telephone survey was carried out between October and November 2022 on a sample of 1002 adult citizens (aged 17 years or older) who were fluent Greek speakers. Data were collected via personal telephone interviews in the participants’ households.

A target sample of 1000 individuals was designed for the purposes of this survey to provide nationally representative results. The target sample was calculated with a ±3% margin of error, 95% confidence level, and 50% response distribution and was proportionately distributed across the 13 administrative regions of the country. A random, stratified, multistage sampling process was employed based on a quota for municipality of residence, gender, and age [32].

In particular, the sample was determined by randomly selecting telephone numbers (landlines) from the National Telephone Company Directory, taking into account geographic region, gender, and age. Stratification was based on the 2021 population-housing census conducted by the Hellenic Statistical Authority [33], and all numbers were categorized by administrative region, prefecture, municipality, and urbanization level. Only telephone numbers belonging to individuals (not businesses or public services) were considered in the study. To achieve the target sample of 1000 participants, a list of approximately 11,000 unique telephone numbers was generated. Multiple contact attempts (at least 5 callbacks) were made. Overall, 9833 conversations were held, out of which 1002 complete interviews were conducted, leading to a response rate of approximately 10.2%.

The survey was performed by a commercial company specializing in the field of demographic surveys, under the monitoring and close guidance of the scientific supervisor of the study. The interviewing process was supported by a computer-assisted telephone interviewing (CATI) technique. Additionally, as a quality control measure, the interviewers’ supervisors would occasionally listen to (parts of) the interviews. The questionnaire was developed in such a way as to ensure the full anonymity of participants. At the beginning of each interview, individuals were informed about the confidentiality of data and the voluntary participation. The interview duration was on average 20 min. No statistically significant difference was observed between respondents and non-respondents in terms of the administrative region. The study was approved by the Research Ethics Committee of the University of Peloponnese.

During the interviews, participants were requested to reflect on the following aspects: (a) health status; (b) healthcare use; and (c) access to healthcare services. The questionnaire consisted of the following sections: (a) participant characteristics, comprising of 23 items, which recorded general socio-demographic and socio-economic data, including age, gender, education, marital status, profession, income level, self-evaluation of financial status, and insurance type; and (b) use and access to healthcare in Greece, comprising of 15 items, which recorded data regarding self-rated health, healthcare utilization, and potential healthcare access barriers to the different types of health services. Healthcare utilization and barriers to accessing healthcare concerned the past 12 months (i.e., the year 2022).

The questionnaire considered different types of items, including close- and open-ended questions, multiple-choice questions, and Likert-type scale items. For instance, self-rated health was measured on a 5-point ordinal scale, including the values very poor, poor, moderate, good, and very good as a response to the question “How would you characterize your health today?” [34]. When asked “What type of health services have you used in the past 12 months?”, participants could choose more than one type (including medical consultation, diagnostic and laboratory tests, hospitalization, and pharmaceuticals). Regarding barriers to accessing healthcare, participants were requested to reply with a yes or no to the question “Have you experienced any obstacles in accessing the health system for a physician visit in the past 12 months?”, and, if yes, they were prompted to choose from a list of potential barriers (e.g., a. No health insurance coverage, b. Delay in scheduling an appointment at a public healthcare facility, etc.) or report any additional obstacles that were not already mentioned.

The questionnaire used in this study has been previously employed to assess citizens’ preferences for primary healthcare reforms in Greece [35]. In this study, the validity and reliability of the questionnaire was pilot-tested on a random sample of 100 individuals, who were requested to assess the questionnaire’s items comprehension using a 5-point Likert scale (with values ranging from 1: I understood nothing, to 5: I understood everything). The pilot study results were subsequently evaluated by a group of experts, and no amendments were deemed necessary [35].

A descriptive analysis was performed to summarize the characteristics of the study participants, as well as the participants’ self-reported health, preferences for healthcare utilization, and perceived barriers in healthcare access. Categorical variables were presented with absolute and relative frequencies. Additionally, the association between the respondents’ income level and the presence of barriers in accessing healthcare services was investigated. Since inquiries regarding the presence of self-reported access barriers for medical consultations, diagnostic tests, hospitalizations, and pharmaceuticals were represented by dichotomous variables, to measure the socioeconomic inequality of such variables, a concentration index (CI), namely, Erreygers’ CI, was calculated for each variable.

The CI is given by the following:

(1) $$CI=\frac{2}{n\mu }\sum_{i=1}^{n}y_{i}R_{i}-1$$

where n is the number of individuals, y  is the variable under study, μ is the mean of the variable under study, and $R_{i}\;$ is the fractional rank. The minimum and maximum values of the CI are −1 and 1, respectively [36].

In the case of bounded variables, such as the variable “self-reported access barriers,” the bounds of the CI depend upon the mean of the variable. In addition, the CI is not scale-invariant and does not satisfy the mirror property, on the basis of which the inequality indices of y and (1−y) have equal absolute values but opposite signs. In these cases, the use of Erreygers’ CIs is recommended. The Errreygers CI is given by the following:

(2) $$Erreygers^{\prime }CI=\;\frac{4\mu }{y^{max}-y^{min}}CI\;$$

where $y^{max}$ and $y^{min}$ are the bounds of the variable under study. Erreygers’ CI lies in the interval [−1, 1]. Negative values indicate that the variable under study is concentrated among the least well-off respondents, whereas positive values indicate that the variable is concentrated among the most well-off respondents [37,38]. The STATA 19 statistical software package was used for the analysis. Specifically, the command conindex was used [39].

## 3. Results

### 3.1. Socio-Demographics

The survey outcomes reflect the complete responses of 1002 participants who were interviewed between October and November 2022. Table 1 presents the demographic and socio-economic characteristics of the total study sample (1002 participants). More than half of the participants were men (51.9%), aged between 40 and 64 years (53.4%), held a tertiary or postgraduate degree (75.6%), and resided in the Attica or Thessaloniki geographical region (53.8%). Around 66% of the participants were married or lived with a partner, while 44% were either retired from work (26.8%) or not working (17.3% including unemployed participants, students, housewives, and men serving the Armed Forces). Just over 45% reported a total (household) monthly income of EUR 1500 or above, approximately 28% reported a monthly income of EUR 1000 or less, while 59.3% self-evaluated their monthly income as low or fairly low. The vast majority of participants (93.6%) were covered by social (public) health insurance, while 6.4% were uninsured or under a welfare benefit scheme, which offers free access to costly pharmaceutical and hospital care in Greece. Given the sufficient insurance coverage, 82% did not report having private insurance, and those who held one used it primarily to cover hospitalization costs (90%).

### 3.2. Healthcare Use

Across the study period, most participants stated a “good” or “very good” health status (67.1%), while the majority (78.5%) reported using health services for a preventive health check at least sometimes in the past 12 months (year 2022) (Table 2). A significant portion of the total sample had used health services over the preceding year (837 out of 1002 participants, 83.5%). Among these, 82.6% of the participants visited a physician, 80.6% had diagnostic/laboratory tests, 63.6% had received pharmaceuticals, and 16.1% had been hospitalized.

Out of the 691 participants who had a medical consultation in 2022, 33.1% preferred visiting a physician working at the NHS, while 31.3% and 30.8% preferred visiting a contracted and non-contracted with EOPYY private physician, respectively. It should be noted that only 3.6% of participants reported visiting a physician contracted by their private insurance company.

With regards to diagnostic/laboratory tests, the majority of participants (75% out of 675) preferred having their diagnostic tests in a private diagnostic laboratory contracted with EOPYY, rather than in public laboratories in NHS hospitals or EOPYY health centers (16.7%) or non-contracted laboratories (4.6%).

On the contrary, 61.5% out of 135 participants who required hospitalization in 2022 preferred to be admitted in a public hospital (NHS, university hospital, or military hospital) rather than in private hospitals or clinics contracted with EOPYY (20%) or their private insurance company (10.4%). Finally, in regards to pharmaceuticals, the vast majority of 532 participants (85.3%) preferred visiting a private pharmacy to obtain medication, followed by 12.4% of participants visiting an EOPYY pharmacy. Only 1.9% of 532 participants reported visiting a public hospital pharmacy for pharmaceuticals. A graphic representation of the participants’ preferences in regards to healthcare utilization for the different types of health services is available in the Supplementary Materials.

### 3.3. Barriers to Access to Health Services

Access to health services in the past 12 months was assessed to highlight potential obstacles in accessing medical consultations, diagnostic/laboratory tests, hospitalization, and pharmaceuticals for the general population in 2022.

Table 3 illustrates that approximately 48% of the participants who had a medical consultation faced barriers in accessing a physician during the last 12 months. Among these, 178 participants were women (49.2%). More participants residing in the Northern and Central Greece and the Aegean–Crete geographical regions self-reported barriers in accessing a medical consultation compared to the geographical regions of Attica and Thessaloniki.

**Figure 1:**
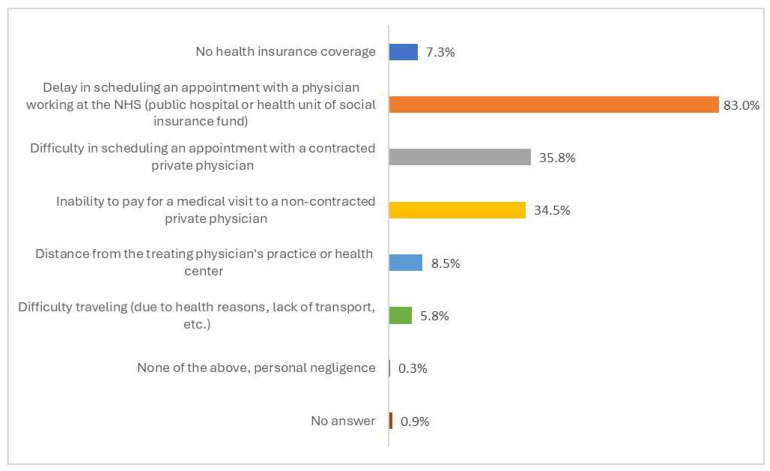
Ranking of barriers in accessing medical consultation in the past 12 months, overall general population (N = 330).

Delay in making an appointment with a physician working at the NHS (public hospital or EOPYY health center) was the most common obstacle encountered in accessing a medical consultation (reported by 83% of the participants) (Figure 1). Difficulties in scheduling an appointment with a private physician contracted with EOPYY and inability to cover the cost of a visit to a non-contracted private physician were also considered important barriers to accessing a medical consultation (35.8% and 34.5%, respectively). Other, less commonly reported obstacles were the geographical distance from the physician’s practice or health center (8.5%), the lack of insurance coverage (7.3%), and difficulties in transportation (5.8%).

The study also revealed that out of 675 participants, 33.8% encountered obstacles in accessing diagnostic and laboratory tests (Table 3). Men and women had similar difficulties in accessing a diagnostic laboratory, while participants with a monthly income of up to EUR 1500 and who resided outside the Attica prefecture experienced barriers at a higher percentage.

**Figure 2:**
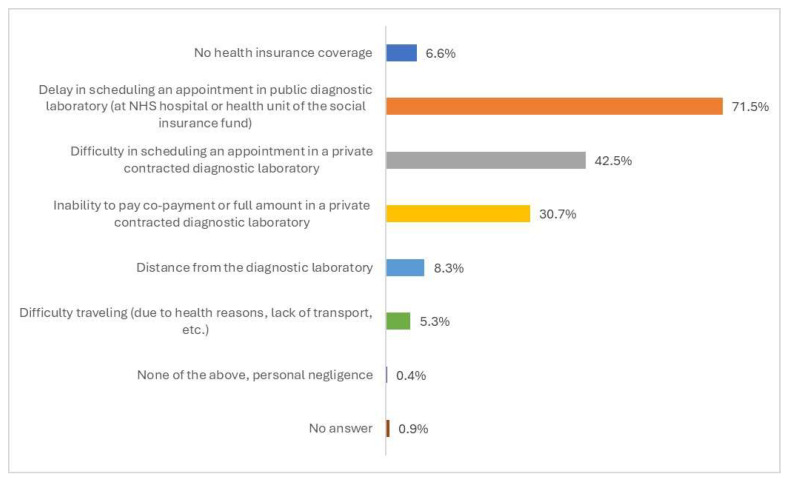
Ranking of barriers in accessing diagnostic and laboratory tests in the past 12 months, overall general population (N = 228).

A total of 71.5% of the participants reported delays in scheduling an appointment at a public diagnostic laboratory (NHS hospital or another EOPYY health center) as the main barrier in accessing diagnostic and laboratory tests (Figure 2). Other frequently reported barriers included challenges in making an appointment with a private diagnostic laboratory contracted with EOPYY (42.5% of the participants) and inability to cover the expenses (part or total cost) of diagnostic and laboratory tests in a contracted private diagnostic laboratory (30.7% of the participants). Barriers due to geographical distance from the diagnostic laboratory (8.3%), lack of insurance coverage (6.6%), and difficulties in transportation (5.3%) were also reported by the participants as barriers in accessing diagnostic and laboratory tests.

A total of 54 participants (40%) answered positively with regard to facing barriers in accessing the health system for a hospitalization (Table 3). Participants with a monthly income of EUR 500 or below experienced barriers in accessing a hospitalization at a considerably higher percentage compared to participants with a monthly income above EUR 2000. Most participants reporting access barriers to hospitalization were between 40 and 64 years old.

**Figure 3:**
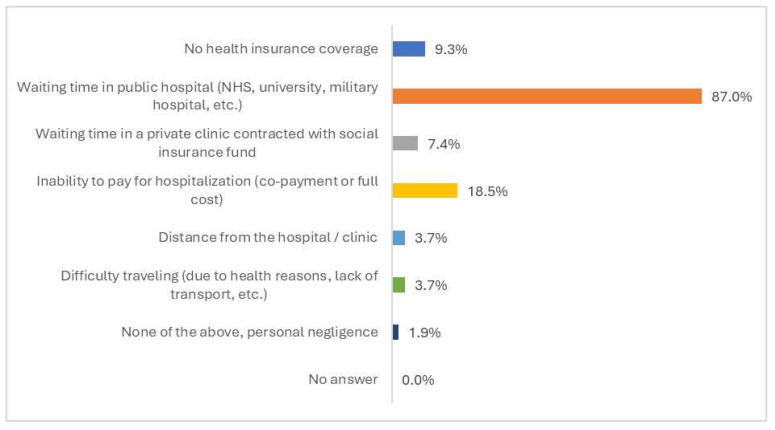
Ranking of barriers in accessing hospitalizations in the past 12 months, overall general population (N = 54).

As shown in Figure 3, the most frequently reported obstacle encountered in accessing hospitalizations was the long waiting time to be admitted to a public hospital (NHS, university or military hospital) (87%), followed by the inability of participants to pay out-of-pocket for a hospitalization in a private clinic (co-payment or full cost) (18.5%). Other, less frequently reported barriers included having no insurance coverage (9.3%), facing long waiting times in a contracted private clinic (7.4%), and distance from the hospital or private clinic (3.7%) (Figure 3).

Barriers in access to pharmaceuticals were reported by 149 participants (28%) (Table 3). Men and women faced similar challenges in accessing pharmaceuticals. Having a monthly income between EUR 1000 and EUR 1500 and residing outside the Attica prefecture were associated with more limited accessibility to pharmaceuticals.

**Figure 4:**
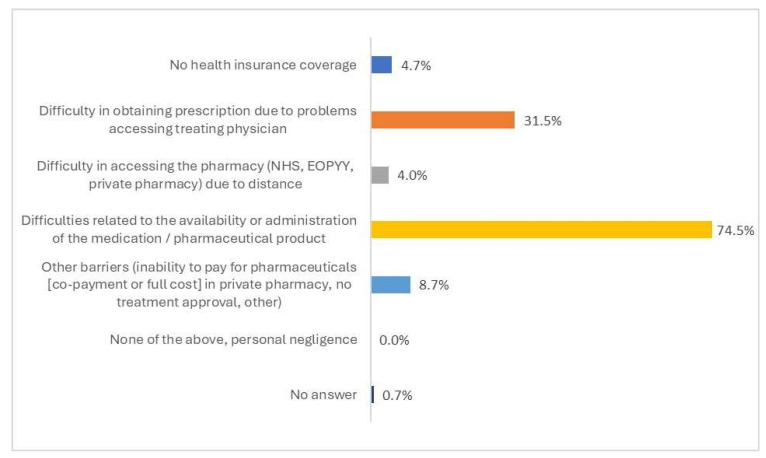
Ranking of barriers in accessing pharmaceuticals in the past 12 months, overall general population (N = 149).

The main perceived barrier in accessing pharmaceuticals was difficulties associated with the availability and/or administration of the respective medication/pharmaceutical product in all types of pharmacies (NHS hospital, private, or EOPYY-affiliated pharmacies) (74.5%) (Figure 4). Other commonly reported obstacles included difficulties in obtaining a medication prescription because of limited access to the treating physician’s practice (31.5%), inability to cover out-of-pocket expenses (co-payment or full cost) for pharmaceuticals in a private pharmacy, lack of treatment approval by the physician, or other obstacles (8.7%).

The statistical analysis performed to measure the socioeconomic inequality of access barriers for the various types of health services yielded the following results: the Erreygers CI for medical consultations was equal to −0.21 (p<0.001); the Erreygers CI for the diagnostic and laboratory tests was found to be −0.14 (p=0.0028); the Erreygers CI for hospitalizations was found to be −0.21 (p=0.0456); and the Erreygers CI for pharmaceuticals was equal to −0.088 (p=0.0742). These results indicate the existence of significant inequalities in self-reported access barriers for medical consultations, diagnostic/laboratory tests, and hospitalization, since the variables under study are concentrated among the least well-off participants in terms of income level.

## 4. Discussion

The present study aimed to assess the public perception of barriers to accessing healthcare services in Greece in 2022. The analysis of the demographic and socioeconomic characteristics of the 1002 survey participants showed that a considerable proportion of the sample belonged to a middle or higher (monthly income of EUR 1000 or above) income class in Greece and did not have private health insurance. Nonetheless, the majority of the participants self-evaluated their monthly income as low or fair to low. Approximately 67% self-evaluated their health status as good or very good, yet the majority of the study participants (83.5%) made use of healthcare services within 2022, following the COVID-19 pandemic era in Greece.

The largest proportion of participants requiring hospitalization relied on social health insurance to cover these services, i.e., they preferred to be admitted to a public hospital. Results also indicated a preference for medical consultations in the public sector (i.e., a physician working at the NHS), although participants showed a preference for outpatient health services in the private sector (contracted or not with EOPYY) to a similar degree. On the other hand, participants’ preference for private diagnostic laboratories with costs covered by public insurance, and private pharmacies for pharmaceuticals far outweighed that for providers in the public sector.

Long waiting lists and high costs were the most commonly identified barriers to accessing healthcare services. Approximately half of the participants requiring a medical consultation experienced issues with access, stemming primarily from long waiting lists for making an appointment with a physician working in a public health facility or a contracted private physician, as well as financial constraints in visiting a non-contracted physician. Similar barriers were identified for diagnostic and laboratory tests and hospitalizations, although financial constraints were more frequently reported as barriers for hospitalization in a private clinic. Difficulties related to the availability or administration of pharmaceuticals and difficulties in obtaining a prescription were considered the most important barriers in accessing pharmaceuticals.

Overall, present findings are in line with previous research regarding barriers in access to healthcare for specific patient groups, such as rheumatoid arthritis patients [29], multiple sclerosis patients [31], and cancer patients [28], which identified long waiting times, and limited financial and physical access as key contributors to barriers to healthcare access. Moreover, our study confirms the findings of prior qualitative surveys, which highlighted high costs and long waiting times for publicly funded health services as major barriers in accessing medical care [12,26,40].

The statistical analysis results also reinforce existing evidence regarding socioeconomic inequalities in healthcare access. In particular, high-income individuals in Greece have consistently been shown to encounter fewer access barriers, also by opting for private services or using informal payments, compared to lower-income individuals who often face long waiting times and geographical constraints [41]. These income-related disparities increased during the COVID-19 pandemic and persisted in the aftermath of the public health crisis. In fact, 27% of the Greek population in the two lowest income quintiles compared to only 4.3% in the highest income quintile reported unmet healthcare needs for medical examination in 2022, while 16.7% of Greek citizens aged 16 years or over self-reported unmet needs for medical consultation due to financial constraints, long waiting times, or geographical distance, evidence which is confirmed by our findings [26,42,43].

Furthermore, the study results indicated similar frequencies of medical consultations across the public and private sectors, which aligns with the persistently increasing reliance on private healthcare within the Greek healthcare system. This was evidenced in the pre-pandemic era following the economic crisis, and was exacerbated following the COVID-19 outbreak disruptions [24,25,41].

This study used a national representative sample of the Greek population, covering all geographical regions of the country, and focused on the time period following a critical public health crisis. However, the sampling process may be a possible study limitation. Eligible participants were only those who had a landline connection, and most households in Greece have access to a fixed telephone; nonetheless, vulnerable populations such as homeless individuals or refugees may have been excluded or under-represented in the study, and these groups often encounter more healthcare access barriers. Other limitations include the potential for response bias, given the self-reported nature of data on healthcare utilization and access barriers, as well as the restriction of the analysis to one country, which may affect the generalizability of results to other country settings.

## 5. Conclusions

Access to healthcare constitutes a major aspect of health policy in Greece since it directly affects population health and potentially contributes to health inequalities. The identified barriers in accessing health services highlight the vulnerabilities of the current health system in Greece, which were further exposed during the COVID-19 pandemic crisis when access to healthcare was significantly hampered. Nonetheless, both the economic downturn in 2008 and the COVID-19 pandemic have served as a catalyst in recognizing the weaknesses of the existing system and fostering healthcare reforms that would otherwise be resisted [44]. Still, based on the findings of the present study, socioeconomic factors may be related to key access (barrier) indicators, such as costs, freedom of choice, and distance. Thus, future health policy initiatives in Greece should focus on understanding and addressing the socioeconomic determinants behind barriers and inequalities in healthcare access.

## Figures and Tables

**Table 1:** Socio-demographic and socio-economic characteristics of the participants.

Demographic and Socio-Economic Characteristics	No. of Respondents	%
**Total population**	1002	100
**Gender**		
Men	520	51.9
Women	482	48.1
**Age (years)**		
≤24	80	8.0
25–39	153	15.3
40–54	280	27.9
55–64	256	25.5
≥65	233	23.3
**Education**		
Basic	24	2.5
Secondary	217	21.7
Higher	495	49.5
Postgraduate (Master’s degree or PhD)	263	26.3
**Geographical area of residence**		
Attica	435	43.4
Thessaloniki	104	10.4
Northern Greece	136	13.6
Central Greece	251	25.0
Aegean–Crete	76	7.6
**Marital status**		
Single	207	21.1
Married/cohabitating	650	66.2
Divorced	90	9.2
Widowed	35	3.5
**Profession**		
Agricultural, fishery worker	9	0.9
Shop and market sales worker	17	1.7
Business owner/businessman	38	3.9
Public sector employee	174	17.8
Private sector employee	181	18.5
Scientist, self-employed, technical assistant	128	13.1
Not working/seeking work for first time	169	17.3
Retired	262	26.8
**Total monthly income (EUR)**		
No income	10	1.1
≤500	39	4.4
EUR 501–1000	196	22.3
EUR 1001–1500	232	26.5
EUR 1501–2000	176	20.0
>2001	226	25.7
**Self-evaluation of monthly income**		
Low/fair to low	588	59.3
Fair	314	31.6
Fair to high/high	90	9.1
**Health insurance status**		
Insured	927	93.6
Not insured/welfare	63	6.4
**Private insurance coverage**		
Yes	176	17.8
No	812	82.2
**Type of services covered by private insurance (if yes**) *		
Physician visit	81	48.5
Diagnostic tests	96	57.5
Hospitalization	150	90.0

* Multiple choice available, total % does not sum up to 100.

**Table 2:** Health status and use of healthcare services (in the past 12 months).

Item	No. of Respondents	%
**Health Status**		
Very bad/bad	90	9.0
Moderate	237	23.9
Good/very good	668	67.1
**Frequency of using health services for preventive health check-up**		
Very often/often	407	40.8
Sometimes	376	37.7
Rarely/never	214	21.5
**Use of health services in the past 12 months**		
Yes	837	83.5
No	165	16.5
**Type of health services used**		
Total	837	100.0
Medical consultation	691	82.6
Dental care visit	376	44.9
Diagnostic and laboratory tests	675	80.6
Hospitalization	135	16.1
Pharmaceuticals	532	63.6

**Table 3:** Self-reported barriers to healthcare access in 2022 for the general population, total sample and by gender, income level, age group, and area of residence.

Self-Reported Access Barriers (Yes/No)	Medical Consultation (n [%])		Diagnostic and Laboratory Tests (n [%])		Hospitalization (n [%])		Pharmaceuticals (n [%])	
	Yes	No	Yes	No	Yes	No	Yes	No
**Total population**	330 (47.8)	331 (47.9)	228 (33.8)	413 (61.2)	54 (40.0)	75 (55.6)	149 (28.0)	349 (65.6)
**Gender**								
Men	152 (46.2)	163 (49.5)	112 (33.9)	201 (60.9)	31 (40.3)	44 (57.1)	72 (27.3)	181 (68.6)
Women	178 (49.2)	168 (46.4)	116 (33.6)	212 (61.4)	23 (39.7)	31 (53.4)	77 (28.7)	168 (62.7)
**Total monthly income (EUR)**								
≤500	24 (70.6)	10 (29.4)	14 (46.7)	16 (53.3)	4 (66.7)	1 (16.7)	10 (40.0)	13 (52.0)
501–1000	71 (53.8)	54 (40.9)	50 (39.7)	69 (54.8)	14 (46.7)	15 (50.0)	29 (26.9)	65 (60.2)
1001–1500	85 (54.1)	69 (43.9)	52 (34.2)	93 (61.2)	13 (46.4)	14 (50.0)	43 (33.9)	80 (63.0)
1501–2000	58 (44.6)	63 (48.5)	46 (36.2)	71 (55.9)	11 (57.9)	8 (42.1)	24 (26.4)	62 (68.1)
>EUR 2001	56 (34.8)	97 (60.2)	40 (24.7)	114 (70.4)	9 (25.7)	24 (68.6)	30 (24.0)	91 (72.8)
**Age (years)**								
≤24	17 (33.3)	31 (60.8)	8 (23.5)	23 (67.6)	3 (50.0)	3 (50.0)	10 (29.4)	18 (52.9)
25–39	33 (34.7)	59 (62.1)	24 (25.3)	65 (68.4)	4 (28.6)	10 (71.4)	22 (39.3)	30 (53.6)
40–54	102 (55.4)	73 (39.7)	75 (41.7)	97 (53.9)	12 (38.7)	16 (51.6)	37 (27.6)	91 (67.9)
55–64	104 (52.5)	87 (43.9)	73 (36.5)	119 (59.5)	25 (51.0)	23 (46.9)	51 (31.3)	106 (65.0)
≥65	74 (45.4)	81 (49.7)	48 (28.9)	109 (65.7)	10 (28.6)	23 (65.7)	29 (20.0)	104 (71.7)
**Geographical area of residence**								
Attica	147 (47.0)	154 (49.2)	93 (30.7)	198 (65.3)	24 (39.3)	34 (55.7)	65 (25.8)	175 (69.4)
Thessaloniki	32 (44.4)	40 (55.6)	25 (34.2)	48 (65.8)	5 (41.7)	6 (50.0)	15 (27.8)	33 (61.1)
Northern Greece	48 (48.5)	47 (47.5)	39 (41.9)	53 (57.0)	6 (54.5)	5 (45.5)	23 (30.3)	50 (65.8)
Central Greece	78 (49.7)	68 (43.3)	52 (33.1)	85 (54.1)	15 (38.5)	22 (56.4)	39 (34.8)	61 (54.5)
Aegean–Crete	25 (50.0)	22 (44.0)	19 (38.8)	29 (59.2)	4 (33.3)	8 (66.7)	7 (18.4)	30 (78.9)

Notes: Refers to 837 participants who required access to healthcare services in the past 12 months.

## Data Availability

The raw data supporting the conclusions of this article will be made available by the authors on request.

## Supplementary Materials

The following supporting information can be downloaded at: https://www.mdpi.com/article/10.3390/healthcare13151867/s1, Figure S1. Medical consultation per provider type (for 691 participants who visited a physician in 2022). Figure S2. Diagnostic/laboratory tests per provider type (for 675 participants who visited a diagnostic laboratory in 2022). Figure S3. Hospitalization per provider type (for 135 participants who were hospitalized in 2022). Figure S4. Pharmaceuticals per provider type (for 532 participants who required medication in 2022).

## Abbreviations

The following abbreviations are used in this manuscript:

EOPYY	National Organization for Health Care Services
EU	European Union
NHS	National Health System
