# Supplementary material for: Barriers in Access to Healthcare Services in Greece Post-COVID-19: Persisting Challenges for Health Policy
Source: Healthcare (Basel). 2025 Jul 30;13(15):1867. doi: 10.3390/healthcare13151867 (PMC12346359; doi:10.3390/healthcare13151867)

Preferences in healthcare utilization per type of health service

Figure S1. Medical consultation per provider type (for 691 participants who visited a physician in 2022).

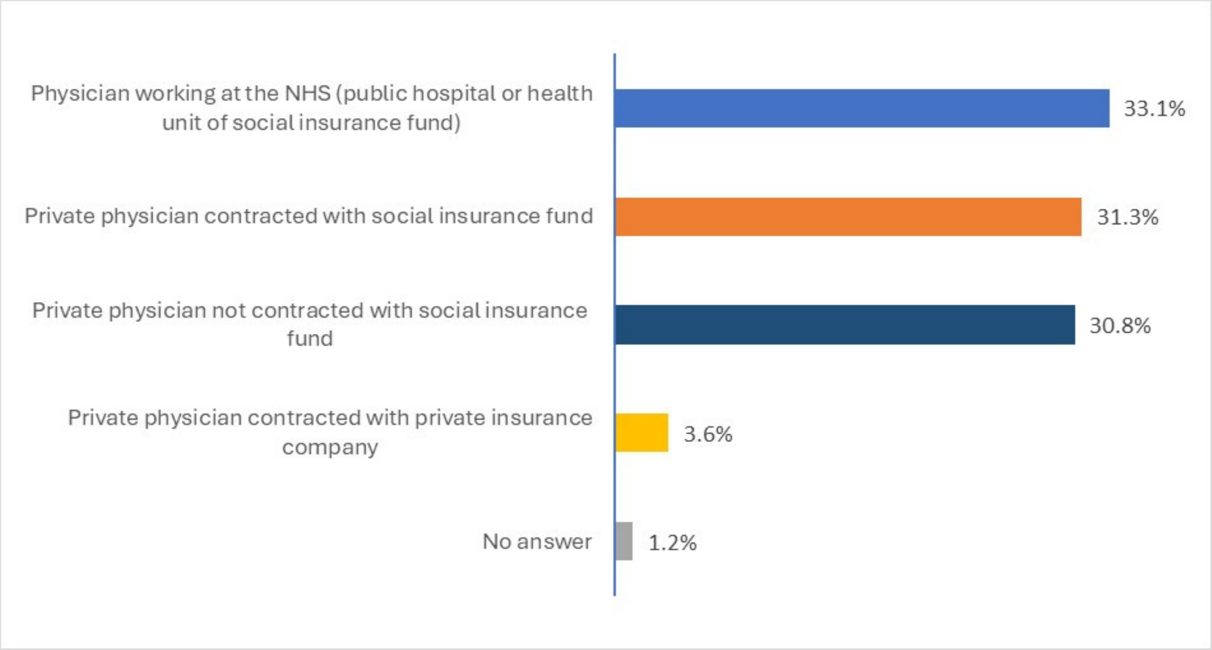

Figure S2. Diagnostic / laboratory tests per provider type (for 675 participants who visited a diagnostic laboratory in 2022).

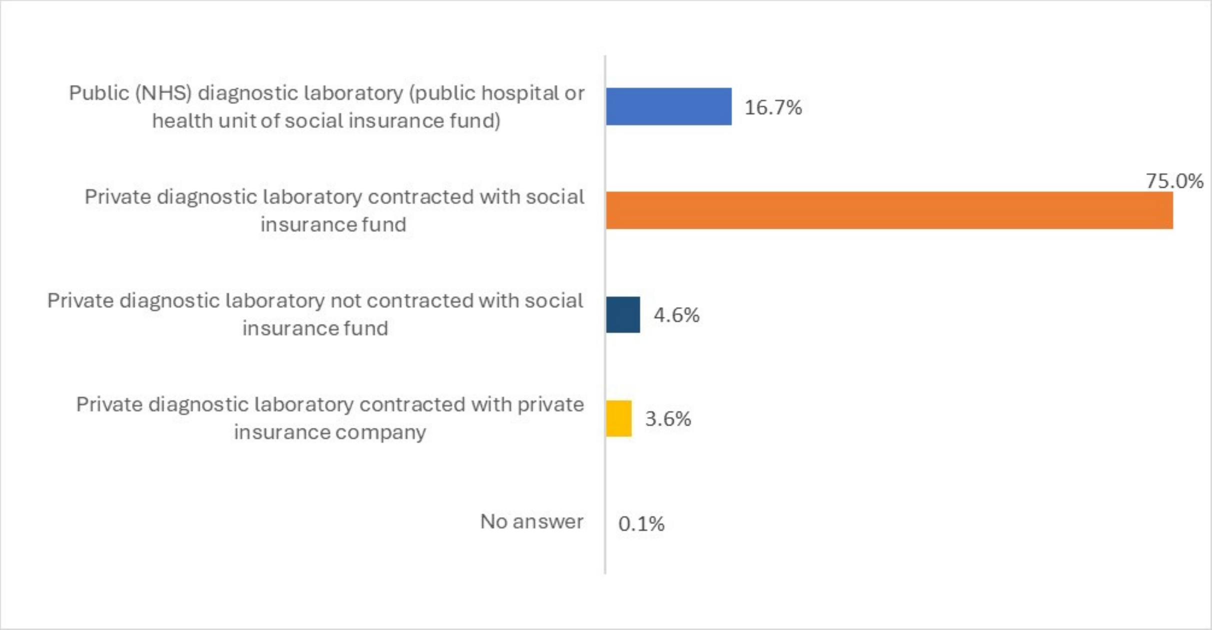

Figure S3. Hospitalization per provider type (for 135 participants who were hospitalized in 2022).

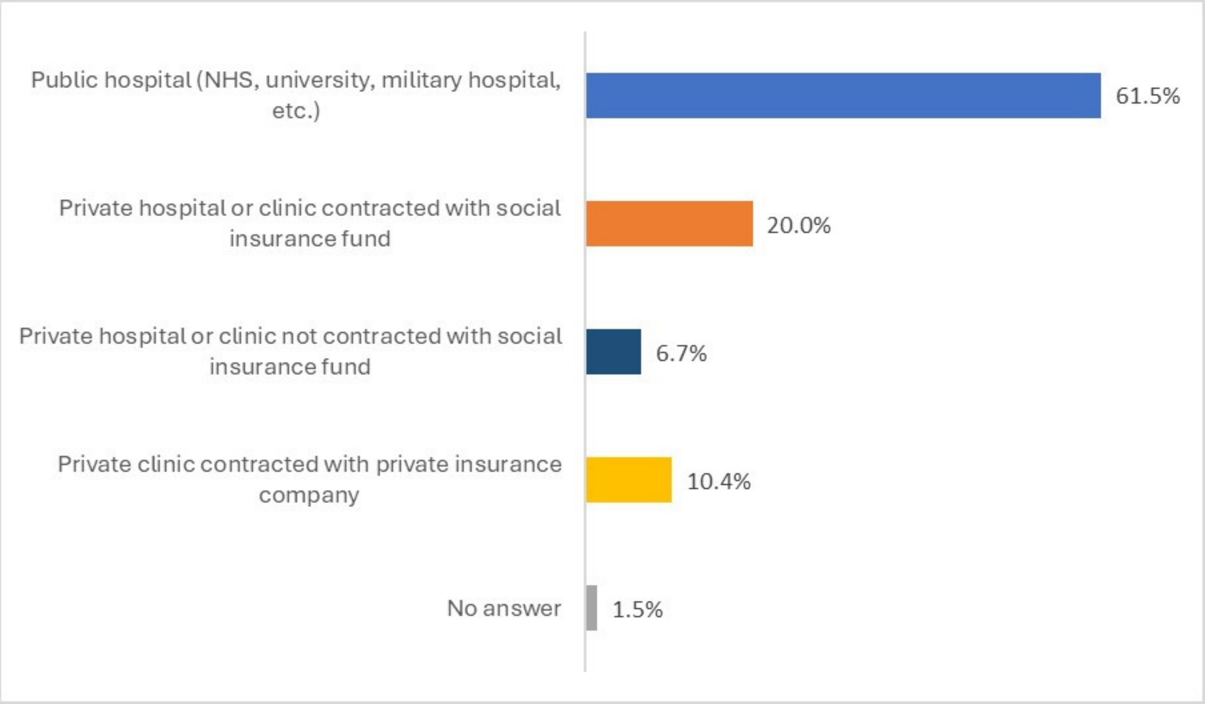

Figure S4. Pharmaceuticals per provider type (for 532 participants who required medication in 2022).

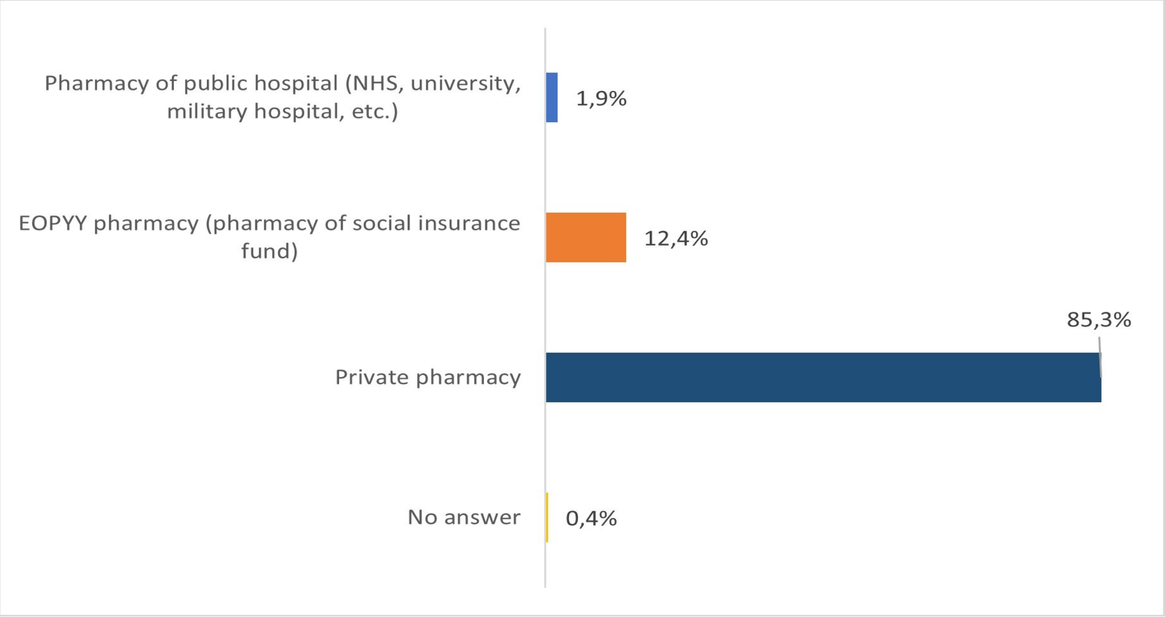

Supplement: Supplementary file 1 [file healthcare-13-01867-s001.zip › healthcare-3698028-supplementary.pdf]
